# Supplementary material for: Electric Cell-Substrate Impedance Sensing (ECIS) as a Platform for Evaluating Barrier-Function Susceptibility and Damage from Pulmonary Atelectrauma
Source: Biosensors (Basel). 2022 Jun 5;12(6):390. doi: 10.3390/bios12060390 (PMC9221382; doi:10.3390/bios12060390)
Supplement: Supplementary file 1 [file biosensors-12-00390-s001.zip › biosensors-1741742-supplementary.pdf]

Supplementary Material

Materials and Method

2.1.4.1. Tested Feeding conditions

Channel Dimension:

Height × Width × length = 0.35 mm × 5.0 mm × 50.0 mm
Number of nodes: 8
Material: Bottom Plate- Glass. Top cover- PET

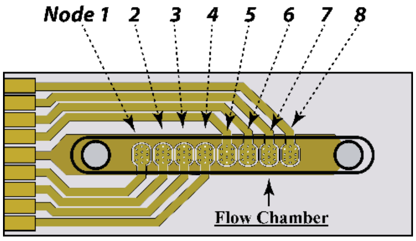

Figure S1: *Schematic of the ECIS flow channel. Eight nodes are numbered from left to right.*

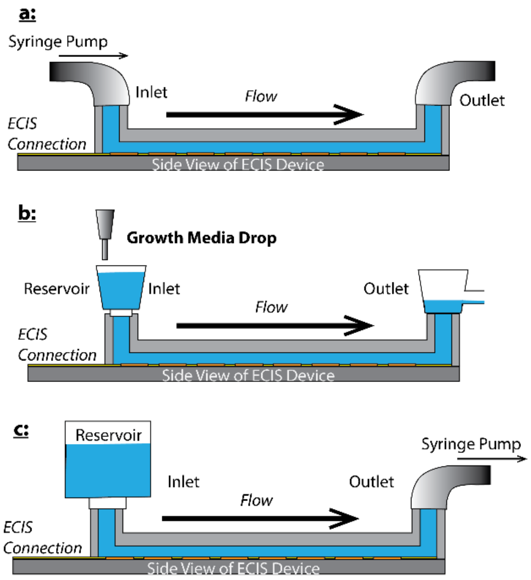

Figure S2 : *Schematics of tested feeding systems.* ***a: Direct feeding from syringe.*** *The growth media was injected through connected tube. Entire hydraulic line was non-air permeable. No open surface. Flexible flow control.* ***b: Droplet system.*** *The growth media was dropped to open surface of the inlet reservoir. The flow was driven by hydraulic pressure generated by gravity. Both side of reservoirs provide open surface to the growth media. Limited flow control.* ***c: Reservoir system.*** *The growth media was pulled from the open inlet reservoir. The inlet reservoir provided open surface to the media. Flexible flow control.*

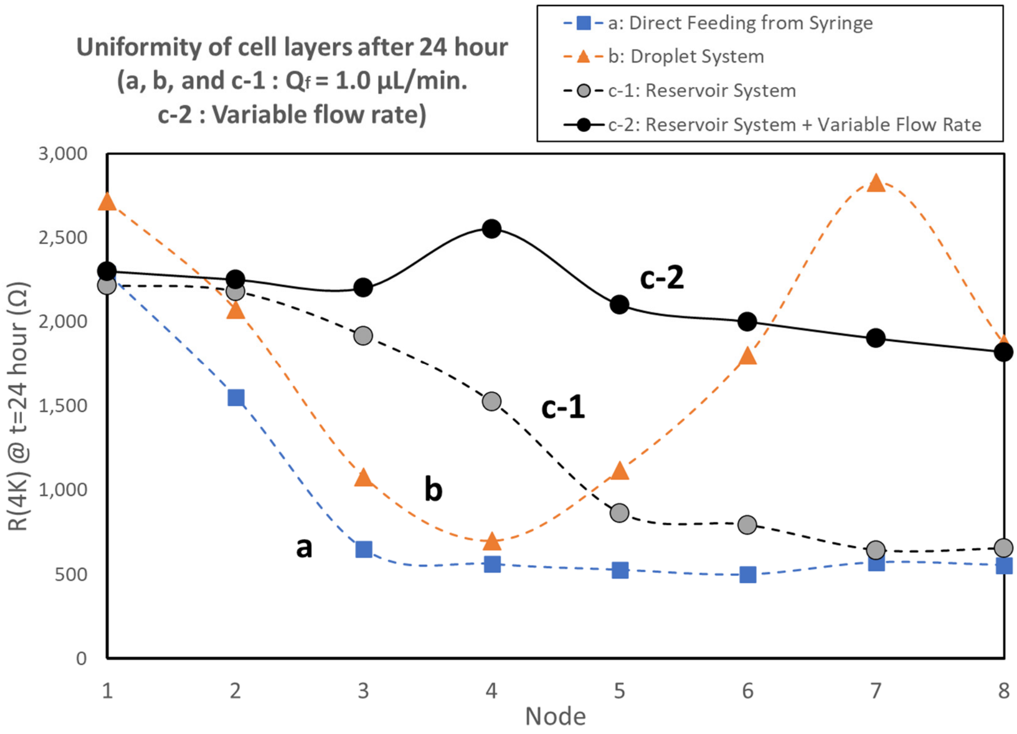

Figure S3: *Uniformity (Resistance at 4k Hz) of H441 layer after 24 hours of incubation.* Q_f *= 1.0 µl/min. is a fixed volumetric flow rate used for a, b, and c-1.* Q_f *is maximum flow rate without impacting the initial settlement of cells.* ***a: Direct Feeding from Syringe*** *with* Q_f*=1.0 µl/min. could not maintain the culture condition after node 3. R(4K) = 500 Ω is a cell-free surface resistance.* ***b: Droplet System*** *has open surfaces on in/outlet ports, however failed to maintain the culture condition due to the limited feeding flow control.* ***c-1: Reservoir System*** *with* Q_f*=1.0 µl/min. failed to maintain the culture condition after node 5.* ***c-2: reservoir System + Variable Flow Rate*** *achieved nearly uniform H441 layer after 24 hours of incubation. This combination allowed us to increase flow rate beyond* Q_f *after the initial cell settlement period.*

Table S1. *Flow rate examples.* ***U*** *is average velocity (µm/sec.) of feeding flow in the channel.* $\boldsymbol{Q_f}$ *is flow rate (µl/min.).* $\boldsymbol{U/d_c}$ *is dimensionless velocity based on diameter of cell (*d_c*). Variable Flow Rate Setting:* Q_f*= 0.2 µl/min. for initial 2 hours,* Q_f*= 0.4 µl/min. for 2 next hours, and* Q_f*= 2.0 µl/min for next 20 hours.*

U/dc	U (µm/sec.)	Qf (µL/min.)
0.05	1	0.105
0.1	2	0.21
0.2	4	0.42
0.5	10	1.05
1	20	2.1
